# Coagulation and Fibrinolysis Indicators and Placental Malaria Infection in an Area Characterized by Unstable Malaria Transmission in Central Sudan

**DOI:** 10.1155/2015/369237

**Published:** 2015-07-30

**Authors:** Amged G. Mostafa, Naser E. Bilal, Awad-Elkareem Abass, Elhassan M. Elhassan, Ahmed A. Mohmmed, Ishag Adam

**Affiliations:** ^1^Faculty of Medical Laboratory Sciences, University of Khartoum, P.O. Box 102, Khartoum, Sudan; ^2^Faculty of Medicine, University of Geziera, Medani, Sudan; ^3^Faculty of Medicine, Ribat University, Khartoum, Sudan; ^4^Faculty of Medicine, University of Khartoum, P.O. Box 102, Khartoum, Sudan

## Abstract

This study aimed to investigate coagulation, fibrinolysis indicators, and malaria during pregnancy. *Methods*. A cross-sectional study was conducted at Medani, Sudan. Sociodemographic characteristics were gathered from each parturient woman (163) and malaria was investigated by blood film and placental histology. Protein C, protein S, antithrombin-III, tissue factor pathway inhibitor (TFPI), and plasminogen activator inhibitor-1 levels (PAI-1) were measured using ELISA. *Results*. One (0.6%), three (1.8), and 19 (11.7%) of the placentae showed active, chronic, and past infection on a histopathological examination, respectively, while 140 (85.9%) of them showed no signs of malaria infection. While the mean [SD] of the protein C, antithrombin-III, and TFPI was significantly lower, there was no significant difference in protein S and PAI-1 levels in women with placental malaria infection (*n* = 23) compared to those without placental malaria infection (140). In linear regression, placental malaria infection was associated with antithrombin-III. There was no association between placental malaria infections and protein C, protein S, TFPI, and PAI-1 levels. There was no association between hemoglobin, birth weight, and the investigated coagulation and fibrinolysis indicators. *Conclusion*. This study showed significantly lower levels of protein C, antithrombin-III, and TFPI in women with placental malaria infections.

## 1. Background

Malaria during pregnancy is a substantial public health problem in endemic tropical countries, especially sub-Saharan Africa. It has been estimated that approximately 125 million pregnant women live in malaria-endemic areas in sub-Saharan Africa and 32 million of these pregnant women are at risk of malaria [1, 2]. Malaria during pregnancy can lead to maternal and fetal adverse effects, mainly anemia and low birth weight (LBW) [3–5]. Pregnant Sudanese women are susceptible to malaria regardless of their age and parity, and malaria is associated with maternal mortality, anemia, LBW, and stillbirth [6–9]. Each year more than 300,000 newborns die from malaria-associated low birth weight [2].

Previous studies indicate that* P. falciparum* malaria especially the severe form can lead to an impairment of the coagulation system which correlates with proinflammatory cytokines [10]. Fibrin deposition is an important feature of placental malaria infections [11, 12]. Furthermore, it has been shown that excessive fibrin deposition in the infected placenta occurs in association with dramatic upregulation of tissue factor, the initiator of the extrinsic pathway of coagulation on infiltrating monocytes [13]. Recently, it has been observed that dysregulated homeostasis is an important feature of placental malaria and anticoagulant treatment (in animal model) may represent a novel therapeutic avenue for averting poor birth outcomes associated with malaria during pregnancy [14]. However, the mechanisms of inflammation, coagulation, and fibrinolysis in the malaria during pregnancy have not been well studied. Thus, the current study was conducted to investigate the coagulation and fibrinolysis indicators protein C, protein S, antithrombin-III, tissue factor pathway inhibitor (TFPI), plasminogen activator inhibitor-1 levels (PAI-1), and placental malaria infection in central Sudan, so as to add to the researches on malaria during pregnancy in Sudan [6–9, 15, 16]. Central Sudan is characterized by unstable malaria transmission.* P. falciparum* is the main malaria parasite species in the area [17].

## 2. Methods

A cross-sectional study was conducted during the rainy and postrainy season of August to December 2011 in the labor ward of the tertiary Hospital (Medani) in the central Sudan. Medani Maternity Hospital is a referral hospital for women who are referred from other health centers and hospitals and women who live close to the hospital facility.

The total sample size was calculated to have over 80% power to detect a difference of 5% at *α* = 0.05, where the expectant prevalence of placental malaria was 25%. We assumed that 10% of women might not respond or have incomplete data.

Apparently, normal women with singleton pregnancy were enrolled to the study. Women with hypertension, diabetes mellitus, and thyroid disease were excluded. After obtaining signed informed consent from women, sociodemographics, history of obstetrics, medical history, antennal attendance characteristics, and bed net use were gathered using structured questionnaires. Body mass index was calculated by measuring maternal weight and height, which was expressed as weight (kg)/height (m)^2^. Newborns were weighed immediately following birth using the Salter scale and the sex of each newborn was recorded.

### 2.1. Giemsa-Stained Blood Smears for Light Microscopy

Maternal, placental, and cord blood films for malaria were prepared, stained by 10% Giemsa. In case of positive films, the number of asexual parasites was counted per 200 leukocytes, assuming a leukocyte count of 8000 leukocytes/*μ*L (for thick films), or per 1000 red blood cells (for thin films). Blood films were considered negative for malaria if no parasites were detected in 100 oil immersion fields of a thick blood film, which was double-checked in a blind manner by an expert microscopist. Maternal hemoglobin concentrations were estimated by the HemoCue hemoglobinometer (HemoCue AB, Ängelholm, Sweden).

### 2.2. Placental Histology

The details of this have been mentioned previously [8, 15, 16]. Briefly, around 3 cm^3^ of placental sample was obtained from the maternal surface, approximately half the distance between the umbilical cord and the edge of the placenta. Then, each biopsy sample was immediately placed in 10% neutral buffered formalin. The buffer was used to prevent the formation of formalin pigment that is similar in its optical characteristics and polarized light activity to malaria pigment [12]. The placental biopsy samples were then processed and were embedded in paraffin wax by standard techniques. In every case, paraffin sections that were 4 mm thick were stained with hematoxylin-eosin and Giemsa stains. Placental malaria infection was classified using histology as previously described by Bulmer et al. as follows [11]: uninfected (no parasites or pigment), acute (parasites in intervillous spaces), chronic (parasites in maternal erythrocytes and pigment in fibrin or cells within fibrin and/or chorionic villous syncytiotrophoblast or strom), and past (no parasites), and pigment confined to fibrin or cells within fibrin. The slides were examined by a pathologist (AMM) who was blind regarding the clinical characteristics of these samples.

### 2.3. ELISA for Measuring Coagulation and Fibrinolysis Indicators Levels

Protein C, protein S, antithrombin-III, TFPI, and PAI-1 levels were measured using a human ELISA kit (Biotain Pharma Co., Ltd., Xiamen City, Fujian Province, China) by following the manufacturer's protocol.

### 2.4. Statistical Analysis

Data were entered into a computer using SPSS for windows (version 16.0). Continuous data (including the above mentioned coagulation and fibrinolysis indicators) were normally distributed and were compared between groups using *t*-test. Multivariate analyses were performed using binary models for placental malaria infection as the dependent variable and linear models with hemoglobin, birth weight, and hemophilia factor levels as continuous dependent variables. Sociodemographic characteristics and placental malaria infections were the independent predictor of interest. Odds ratios (OR) and 95% confidence intervals (CI) were calculated and a *P* value of <0.05 was considered significant.

### 2.5. Ethics

The study received ethical clearance from the Research Board at the Faculty of Medicine, University of Khartoum, Sudan.

## 3. Results

One hundred sixty-three women were enrolled in the study, 65 (39.8%) were primiparae, and 79 (48.5%) had rural residency. Two (1.2%), 47 (28.8%), and 114 (69.9%) of these 163 women had no, one to two visits, and more than two visits of antenatal care, respectively. The majority (149; 91.4%) of them used bed nets during the index pregnancy. None of these women gave history of using intermittent preventive treatment (IPT).

The mean (SD) hemoglobin level was 10.5 (1.1) g/dL, and 72 (57.1%) of the women were anemic (hemoglobin <11 g/dL). Thirty-eight (23.3%) of these 163 women had blood group A, 19 (11.7%) had blood group B, three (1.8%) had blood group AB, and 103 (63.2%) had blood group O. The mean (SD) of the birth weight was 3132.1 (5169.4), 16 (9.8%) women delivered low birth weight neonates (<2500 g), and 12 had preterm (<37 weeks of gestational age) delivery.

### 3.1. Malaria Infections

There were no* P. falciparum*-positive blood films from maternal peripheral blood, the placenta, or cord blood samples. One (0.6%), three (1.8), and 19 (11.7%) of the placentae showed active, chronic, and past infection on a histopathological examination, respectively, while 140 (85.9%) of them showed no signs of malaria infection. There were no significant associations between age, parity, residence, antenatal care, blood group, and placental malaria infection (Table 1).

While there was no significant difference in the hemoglobin [10.4 (1.1) versus 10.7 (1.3) g, *P* = 0.331] and birth weight [2945.5 (6432.6) versus 3162.0 (4899.2) g, *P* = 0.068], the gestational age [37.7 (1.7) versus 38.8 (1.8) weeks, *P* = 0.012] was significantly lower in women with placental malaria infection (*n* = 23) compared to women who did not have placental malaria infection. Likewise in linear regression, placental malaria infection was not associated with hemoglobin (−0.822 g, *P* = 0.439) and birth weight (0.147 g, *P* = 0.432) (Table 2).

### 3.2. Coagulation and Fibrinolysis Indicators

**Figure 1:**
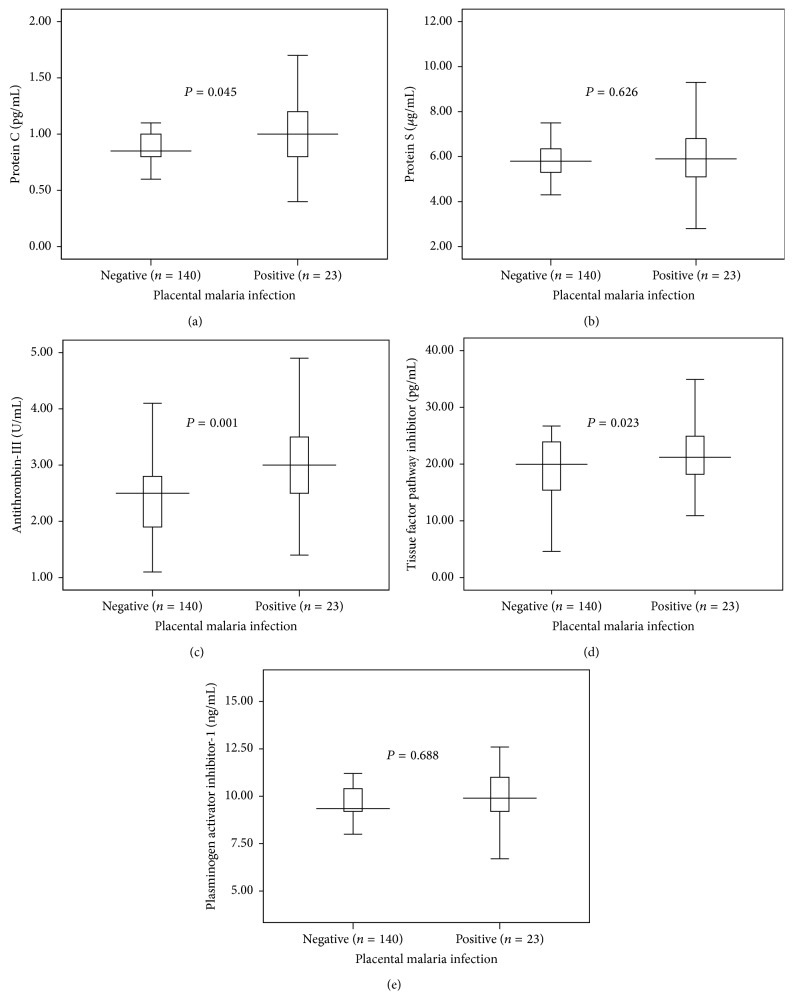


While the mean [SD] of the protein C, antithrombin-III, and TFPI was significantly lower, there was no significant difference in protein S and PAI-1 levels in women with placental malaria infection (*n* = 23) “compared to those without placental malaria infection (*n* = 140), Table 2, Figure 1.”

Similarly, antithrombin-III, TFPI, and PAI-1 levels were significantly lower in women with blood group O than in those who had blood groups other than O (Table 3).

In linear regression, placental malaria infection was associated with antithrombin-III (−4.091 mg, *P* < 0.001). There was no association between placental malaria infections and protein C, protein S, TFPI, and PAI-1 levels. There was no association between hemoglobin, birth weight and protein C, protein S, antithrombin-III, TFPI, and PAI-1 levels (Table 4).

In linear regression, there was a significant association between protein S, PAI-1, and protein C. Likewise, there was significant association between PAI-1-1, TFPI, and antithrombin-III (Table 5).

## 4. Discussion

The main findings of the current study were as follows: placental malaria affects women regardless of their age and parity. While the levels of protein S and PAI-1 were not different, the levels of protein C, antithrombin-III, and TFPI were significantly lower in women with placental malaria infection than in those without placental malaria infection.

The lack of association between age, parity, and placental malaria infection in the current study goes with the previous observations where pregnant women in the different regions of Sudan are susceptible to the peripheral, placental, and submicroscopic malaria regardless of their age or parity [6, 8, 15, 16]. Most of the regions of Sudan are characterized with unstable malaria transmission and this could explain the lack of age/parity and malaria infections [17].

It has recently been shown that both active coagulation and suppressed fibrinolysis are evident at the placental level in association with malaria infection in primigravidae women [14]. Therefore, based on the later findings [14], anticoagulant treatment may represent a novel therapeutic option to avert the poor birth outcomes associated with malaria in animal model [14].

Poovassery and Moore observed that in malaria infected placentae macrophages accumulated and expressed tissue factor, and these macrophages tissue factor expressions in placentae could explain low birth weight in malaria placental infection [18]. In placental malaria infections, monocytes/macrophages and endothelial cells express tissue factor by cytokines that can mediate inflammation; cytokines are elevated in placental malaria infections and are associated with placental insufficiency and with low birth weight [19]. It is worth mentioning that cytokines (including IFN-gamma) were significantly elevated and monocytes/macrophage were detected in higher rates in placentae with malaria infections than in uninfected ones in the same area of the study [15, 16]. Therefore, these findings are supported with previous literature that describes the “inflammation-coagulation process” in severe malaria as well as in other diseases such as bacterial sepsis [20, 21].

Interestingly, the “inflammation coagulation cycle” has been observed in preeclampsia which is another health problem during pregnancy and associated with significant maternal morbidity and poor birth outcomes [22]. We have recently observed that women with placental malaria in the same hospital were 2.3 times at higher risk of preeclampsia than women who had no placental malaria infections [23].

There was no association between hemoglobin, birth weight, and the investigated coagulation and fibrinolysis indicators in the current study. Perhaps submicroscopic placental malaria, which was not investigated in the current study rather than histology detected malaria, is the main determinant of low birth weight in this setting [8]. Both microscopic malaria and submicroscopic malaria were reported to activate coagulation and suppressed fibrinolysis in primigravidae women [14]. In the current study, one patient with acute placental malaria infection was missed/undiagnosed by the microscopist. Perhaps the malaria parasite sequestrated in the intervillous space and was difficult to be detected via the microscope.

Although there was no association between the blood group O and placental malaria infection in the current study, antithrombin-III, TFPI, and PAI-1 levels were significantly lower in women with blood group O. Previous reports showed an increased risk of placental malaria in women with blood type O [24] that was recently contradicted by the reverse finding where there was a lower prevalence of placental malaria in primiparae with blood group O [25].

## Figures and Tables

**Table 1:** Univariate and multivariate analyses of factors associated with placental malaria infection.

Variable	Univariate analysis			Multivariate analysis		
	OR	95% CI	*P*	OR	95% CI	*P*
Age	1.0	0.9–1.1	0.292	1.0	0.9–1.1	0.534
Primiparae	1.1	0.4–2.9	0.185	1.5	0.4–5.0	0.482
Residence	1.1	0.4–2.8	0.701	0.8	0.2–3.0	0.773
Maternal education < secondary level	1.5	0.6–4.1	0.340	1.1	0.3–3.7	0.867
Lack of antenatal care	1.6	0.6–4.0	0.309	1.5	0.3–6.6	0.584
Body mass index	1.0	0.8–1.2	0.851	1.0	0.8–1.3	0.766
Hemoglobin	0.8	0.5–1.2	0.329	0.6	0.4–1.0	0.103
Blood group O	0.6	0.2–1.8	0.457	0.8	0.3–2.5	0.804

**Table 2:** Comparing the mean (SD) of the coagulation and fibrinolysis indicators in women with placental malaria infection and controls.

Variable	Placental malaria infection positive (*n* = 23)	Placental malaria infection negative (*n* = 140)	*P*
Protein C, pg/mL	0.9 (0.3)	1.0 (0.2)	0.045
Protein S, *µ*g/mL	5.8 (1.3)	6.0 (1.4)	0.626
Antithrombin-III, U/mL	2.4 (0.6)	3.0 (0.7)	0.001
Tissue factor pathway inhibitor, pg/mL	18.5 (7.0)	21.4 (5.2)	0.023
Plasminogen activator inhibitor-1, ng/mL	9.5 (1.9)	9.8 (1.8)	0.688

**Table 3:** Comparing the mean (SD) of the coagulation and fibrinolysis indicators in women with blood group O.

Variable	Women with blood group O (*n* = 103)	Women with blood group other than O (*n* = 60)	*P*
Protein C, pg/mL	0.9 (0.3)	0.9 (0.2)	0.977
Protein S, *µ*g/mL	5.9 (1.5)	6.1 (1.3)	0.514
Antithrombin-III, U/mL	2.8 (0.7)	3.1 (0.6)	0.020
Tissue factor pathway inhibitor, pg/mL	19.7 (5.7)	22.6 (4.7)	0.016
Plasminogen activator inhibitor-1 ng/mL	9.4 (1.9)	10.1 (1.7)	0.019

**Table 4:** Linear regression analysis of factors associated with maternal hemoglobin and birth weight.

Variable	Maternal haemoglobin			Birth weight		
	Coefficient	SE	*P*	Coefficient	SE	*P*
Age	0.022	0.035	0.525	0.025	0.014	0.081
Primigravidae	0.705	0.386	0.073	0.367	0.155	0.021
Residence	0.215	0.395	0.588	−0.156	0.164	0.346
Maternal education < secondary level	0.011	0.360	0.976	0.157	0.150	0.302
Lack of antenatal care	0.376	0.444	0.401	−0.157	0.180	0.387
Body mass index	−0.064	0.075	0.397	0.005	0.030	0.871
Blood group O	0.162	0.319	0.614	0.218	0.130	0.099
Placental malaria infection	−0.822	0.439	0.066	0.147	0.186	0.432
Hemoglobin	—	—	—	−0.006	0.055	0.915
Protein C	0.288	0.104	0.840	1.0	0.586	0.080
Protein S	−0.356	0.202	0.083	−0.056	0.086	0.521
Antithrombin-III	0.413	0.413	0.322	0.072	0.178	0.688
Plasminogen activator inhibitor-1	0.139	0.104	0.186	0.002	0.044	0.962
Tissue factor pathway inhibitor	−0.018	0.042	0.673	−0.023	0.017	0.198

**Table 5:** Linear regression analysis of factors (including placental malaria) associated with coagulation and fibrinolysis indicators in Medani Hospital.

Variable	Protein C		Protein S		Antithrombin-III		Tissue factor pathway inhibitor	
	Coefficient	*P*	Coefficient	*P*	Coefficient	*P*	Coefficient	*P*
Age	0.004	0.288	0.032	0.178	0.001	0.979	0.147	0.267
Primigravidae	−0.008	0.523	0.084	0.301	−0.189	0.156	0.270	0.855
Body mass index	0.007	0.321	0.036	0.433	0.053	0.035	0.074	0.782
Blood group O	−0.027	0.390	0.058	0.774	−0.250	0.037	−3.626	0.002
Placental malaria infection	−0.007	0.874	−0.218	0.473	0.507	0.005	2.979	0.091
Hemoglobin	−0.002	0.873	0.151	−0.061	0.070	0.167	−0.130	0.790
Protein C	—	—	—	—	—	—	—	—
Protein S	2.982	0.001	—	—	—	—	—	—
Antithrombin-III	0.007	0.846	0.472	0.051	—	—	—	—
Plasminogen activator inhibitor-1	0.025	0.015	0.052	0.432	0.067	0.030	1.177	0.001
Tissue factor pathway inhibitor	−0.005	0.248	0.031	0.212	0.052	0.001	—	—
